# Variations in NT-proBNP Levels in Pregnant Patients with and without SARS-CoV-2 Infection: Consequences for the Newborns

**DOI:** 10.3390/biomedicines11112964

**Published:** 2023-11-02

**Authors:** Carmen-Ioana Marta, Marius Craina, Razvan Nitu, Anca Laura Maghiari, Simona-Alina Abu-Awwad, Lioara Boscu, Mircea Diaconu, Catalin Dumitru, Bogdan Sorop, Lavinia Stelea, Katalin Babes

**Affiliations:** 1 Faculty of Medicine and Pharmacy of Oradea, University of Oradea, 410087 Oradea, Romania; silaghi.carmen@gmail.com (C.-I.M.); piszekati@yahoo.co.uk (K.B.); 2 Clinic of Obstetrics and Gynecology, “Pius Brinzeu” County Clinical Emergency Hospital, 300723 Timisoara, Romania; mariuscraina@hotmail.com (M.C.); diaconu.mircea@umft.ro (M.D.); dumitru.catalin@umft.ro (C.D.); bogdan.sorop@umft.ro (B.S.); stelea.lavinia@umft.ro (L.S.); 3 Department of Obstetrics and Gynecology, Faculty of Medicine, “Victor Babes” University of Medicine and Pharmacy, 300041 Timisoara, Romania; 4 Department I—Discipline of Anatomy and Embryology, “Victor Babes” University of Medicine and Pharmacy, 300041 Timisoara, Romania; boscu.anca@umft.ro; 5 Doctoral School, “Victor Babes” University of Medicine and Pharmacy, 300041 Timisoara, Romania; alina.abuawwad@umft.ro (S.-A.A.-A.); lioara.boscu@umft.ro (L.B.); 6 Clinical County Emergency Hospital of Oradea, 410167 Oradea, Romania

**Keywords:** NT-proBNP, SARS-CoV-2, pregnancy, COVID-19, low birth weight, birth outcomes, neonatal health

## Abstract

Background: NT-proBNP (N-terminal pro-brain natriuretic peptide) has a high negative predictive value for ruling out heart failure, a disorder linked with volume overload of the ventricles, and is used for diagnosis, prognosis, and risk stratification. Pregnancy causes in healthy women changes in physical and hemodynamic parameters and appears to be a risk factor for severe COVID-19 illness. The purpose of this study is to highlight the significance of monitoring NT-proBNP levels during pregnancy, particularly in patients who were infected with COVID-19 during pregnancy or were infected with the virus while giving birth. The findings of this comparative research indicate the importance of NT-proBNP in terms of foetal prognosis and birthweight. Methods: We collected blood samples to measure NT-proBNP concentrations from a cohort of 186 pregnant patients divided into two groups based on the presence of SARS-CoV-2 viral infection. Results: Elevated NT-proBNP had an unfavourable implication on the newborn birth weight and Apgar score and expressed its influence on lower values. Conclusions: We consider that NT-proBNP testing in pregnant patients, especially those with COVID-19 infection, can be relevant and be used as a predictive marker to be taken into consideration when it comes to management, outcome, and treatment regarding pregnant patients and their newborns.

## 1. Introduction

Pregnancy causes volume overload and alterations to the circulatory systems [1,2]. In healthy women, it also causes changes in physical and hemodynamic parameters [3,4]. NT-proBNP (N-terminal pro-brain natriuretic peptide) has a high negative predictive value for ruling out heart failure, a disorder linked with volume overload of the ventricles, and is utilised for diagnosis, prognosis, and risk stratification [5]. During a normal pregnancy, the increase in plasma volume and renal excretion may impact the levels of heart biomarkers used to rule out heart failure (NT-proBNP) [6]. When it comes to identifying heart failure in pregnant patients, NT-proBNP is frequently measured. According to certain research, elevated NT-proBNP levels in early pregnancy are associated with an increased risk of developing severe pregnancy outcomes, such as preeclampsia [7]. Pregnancy appears to be a risk factor for severe COVID-19 illness [8]. Among the most significant modifications to normal physiologic processes that occur during pregnancy are changes to the immune system to accommodate the growing and developing foetus and preserve the pregnancy. The body’s ability to modify the immune system usually provides a balance between the foetus’s growth and development and the ability to fight off invading viruses. Throughout the early stages of pregnancy, the immune system is proinflammatory with immune cells present near the implantation site to support the embryo and placental growth. The maternal immune system and the trophoblasts of the foetal-placental unit actively secret anti-inflammatory cytokines that remove dying trophoblastic cells and protect foetal cells once the embryo is nidated. This protects the foetus from the mother’s immune system and supports its growth and development. The immune system becomes proinflammatory again throughout the third trimester in order to initiate and maintain labour and aid placental separation after birth. These modifications render pregnancy more prone to COVID-19 infection and its associated hyperinflammatory state [9]. Although in severe acute respiratory syndrome coronavirus 2 (SARS-CoV-2), intrauterine transmission appears to be uncommon, most studies demonstrate that COVID-19 during pregnancy increases the risk of pregnancy problems, with individuals with severe forms of disease having a higher risk than those with mild forms of disease [8]. SARS-CoV-2 infection increases the probability of adverse pregnancy outcomes, such as premature birth and stillbirth. There is also rising concern about the consequences of SARS-CoV-2 infection on the placenta, and these effects appear to differ between virus types [10]. There are currently no acknowledged reference values for NT-proBNP in pregnant women, especially those infected with SARS-CoV-2, making it difficult to assess the high values of NT-proBNP and their severity prognosis [11]. Despite the fact that SARS-CoV-2 appears to be rarely transmitted transplacentally to the foetus, evidence suggesting SARS-CoV-2 infection during pregnancy is related with a number of unfavourable pregnancy outcomes is concerning [12]. Preeclampsia, premature birth, gestational diabetes, and low birthweight were associated with severe COVID-19 forms of disease in pregnant women compared to those with mild form of disease [13]. Adverse pregnancy and newborn outcomes are more common in SARS-CoV-2-infected pregnant women, especially those with severe forms of the viral infection [12]. Low birth weight (LBW) is a major public health concern in low- and middle-income countries [14,15]. Intrauterine growth restriction (IUGR) is a major and silent cause of illness and mortality in the foetal and neonatal populations, and it is characterised as a foetal development rate that is slower than normal for the foetal growth potential. IUGR is caused by a number of causes, including maternal, placental, and foetal factors, as well as recently added genetic factors [16]. The purpose of this study is to highlight the significance of monitoring NT-proBNP levels during pregnancy, particularly in patients who were infected with COVID-19 during pregnancy or are infected with the virus while giving birth. The findings of this comparative research of NT-proBNP levels in pregnant patients with or without SARS-CoV-2 infection indicate the importance of this biomarker in terms of foetal prognosis and birthweight. Furthermore, our findings have practical implications for managing pregnant women with high values of NT-proBNP associated with SARS-CoV-2 infection in order to assess the case progression and prognosis.

## 2. Materials and Methods

### 2.1. Study Population/Sample Selection

In the present study, considering NT-proBNP variations in pregnant patients with and without SARS-CoV-2 infection and its consequences for the newborn, we collected blood samples to measure NT-proBNP concentrations from a cohort of 186 pregnant patients. Methodologically, based on the SARS-CoV-2 diagnostic outcomes, the cohort was divided into two distinct groups. Group 1 included 99 pregnant participants with no detectable SARS-CoV-2 infection and normal to elevated NT-proBNP levels, whereas Group 2 included 87 pregnant participants confirmed with SARS-CoV-2 viral infection throughout pregnancy with normal to elevated NT-proBNP levels. All NT-proBNP values above 70 pg/mL were taken into consideration in our study.

This observational study was methodically performed in Timișoara’s “Pius Brînzeu” County Emergency Hospital from 1 January 2021 to 31 December 2022.

### 2.2. RT-PCR SARS-CoV-2 Analysis

The nasopharyngeal and oropharyngeal swabbing techniques were used to obtain samples for the identification of the SARS-CoV-2 viral infection. A medical professional, using suitable infection-control equipment, used a specific swab, introducing it into the patient’s nostril, reaching the nasopharynx, and then rotating it several times to achieve adequate epithelial cell collection. This process was repeated through the mouth in the oropharynx with a second swab. The used swabs were then packed in a sterile viral transport medium to keep the viral particles viable for transfer to the testing facility.

To detect and quantify the SARS-CoV-2 virus in the analysed samples, the reverse transcription polymerase chain reaction (RT-PCR) was used. The viral RNA is initially converted into complementary DNA (cDNA) using a reverse transcription enzyme. The DNA is then exponentially expanded using a thermostable polymerase. Because specific probes and primers are used, the viral target can be quantified and detected precisely. The virus is confirmed by recognising specific fluorescent signals released throughout the amplification process.

### 2.3. NT-proBNP Analysis

The collection of venous blood samples was performed using a conventional venipuncture technique in order to determine the concentration of NT-proBNP in the patients included in our cohort. After confirming the patient’s wellbeing and applying a tourniquet, a vein, usually in the antecubital fossa, was detected. Venous blood was extracted into a syringe or collection tube after a sterile needle was inserted into the vein. The blood samples were obtained and brought to the laboratory, where they were centrifuged to separate the plasma from the blood cells. The presence of NT-proBNP in the plasma was then determined using an immunological technique known as a solid-phase enzyme-linked immunosorbent assay (ELISA). This test employed specific antibodies that bind to NT-proBNP, enabling the accurate detection and quantification of this biomarker.

#### Inclusion and Exclusion Criteria

Participants were carefully chosen in accordance with the study’s well-defined inclusion and exclusion criteria.

Inclusion criteria:

- Patients between 18 and 45 years old;
- Patients were willing to voluntarily participate and provide blood samples for analysis;
- Patients who were tested positive for the SARS-CoV-2 infection confirmed by the RT-PCR method throughout pregnancy;
- Patients who did not have a history of the SARS-CoV-2 infection throughout pregnancy;
- Patients who were tested positive for SARS-CoV-2 infection confirmed by the RT-PCR method at the moment of delivery;
- Pregnant women who gave birth in our institution (vaginal or caesarean section delivery);
- Women without known pre-existing cardiovascular diseases or other conditions that might influence NT-proBNP levels;
- Pregnant patients who signed the informed consent for our study;
- Women who benefited from prenatal care and had regular check-ups during pregnancy.

Exclusion criteria:

- Patients who have had major complications during pregnancy, such as severe preeclampsia, uncontrolled gestational diabetes, or pregnancy-induced hypertension;
- Women who are not pregnant or who are postpartum;
- Pregnant patients who had a history of drug abuse;
- Patients who have taken medications that might influence NT-proBNP levels during pregnancy;
- Women who have a history of psychiatric disorders;
- Women who are unwilling to participate in the study and have not signed the informed consent;
- Patients who have a history of adverse reactions to blood draws;
- Patients who have been enrolled in other clinical trials in the past 9 months;
- Pregnant patients who did not perform regular check-ups during the pregnancy.

### 2.4. Ethical Considerations

This study followed strict ethical guidelines. All participants provided informed consent prior to any data or blood collection after being sufficiently informed about the study’s objectives, methodology, potential risks, and benefits. The study protected the anonymity and confidentiality of the participants’ information by removing or sufficiently encrypting personal patient identification information. Ethical approval was obtained from the institutional review board (approval No.: 6/15 January 2021) prior to commencement. Any concerns highlighted by the participants were addressed throughout the study, ensuring that their rights and well-being were consistently respected.

In their informed consent, participants in this study were well-informed about the study’s goal, which is to elucidate any fluctuations in NT-proBNP levels during pregnancy, as well as the potential modifications to these levels caused by SARS-CoV-2 infection and the impact on the newborn. Participants were instructed about the procedures, which primarily involved blood drawing, as well as potential mild consequences, such as transient discomfort at the puncture site. Their autonomy, anonymity, and option to withdraw from the study at any point without endangering their standard treatment were all fulfilled.

### 2.5. Statistical Analysis

The data used in this study went through a data processing phase using the GraphPad Prism software, notably version 5. The primary method for summarising the data was descriptive statistics, with group comparisons carried out using *t*-tests. It should be noted that all statistical tests performed during the course of this study were two-tailed, and any *p* values less than 0.05 were considered statistically significant. The study results were then given as mean values with their standard deviations (expressed as mean standard deviation, or SD).

We also used the z-test for binomial proportions to differentiate the observed percentages of the two cohorts. This statistical method is appropriate for comparing proportions from two distinct groups, particularly when dealing with categorical data, as evidenced by our cohorts’ demographic information. The purpose of this test is to see if there is a statistically significant difference between the properties of the two groups. The analytical procedure begins with the calculation of a z-statistic, which is then compared to a standard normal distribution in order to calculate the corresponding *p* value. A small *p* value implies a more obvious difference between the proportions, revealing whether the observed variation is related to random chance or represents a real difference between the studied groups.

## 3. Results

We initiated our study with a cohort of 186 pregnant patients. Their ages ranged between 18 and 45, with an average age of 29.3 ± 6.2 years (Table 1). There were no statistically significant differences between the groups in terms of demographics (Table 2).

Detailed comparisons between individuals with and without SARS-CoV-2 are presented in Table 2. Age, body mass index (BMI), reproductive history, and the number of antenatal care (ANC) visits exhibited minor differences between the groups, with their respective *p* values suggesting no statistically significant disparities. Similarly, education levels and living environment, whether urban or rural, were distributed comparably across both categories.

However, a notable variation becomes apparent in the occupation category. A higher proportion of individuals with SARS-CoV-2 were identified as unemployed or skilled workers compared to their counterparts. Conversely, a larger segment of the group without the SARS-CoV-2 viral infection had a professional occupation. The corresponding *p* values confirm the statistical significance of these occupational disparities.

Comparisons between the associated comorbidities of the patients in relation to the status of the SARS-CoV-2 infection were not statistically significant, except for bronchial asthma, which was predominantly found in Group 1 (Table 3). However, these associated pathologies had no statistical influence on the study’s outcome.

In Group 1, the NT-proBNP levels had an average value of 88.7 pg/mL ± 24.8. Conversely, for Group 2, the average NT-proBNP level was found to be 542.3 pg/mL ± 429.5. Using a *t*-test, this difference was found to be statistically significant (*p* < 0.05) (Table 4), indicating that SARS-CoV-2 infection during pregnancy might be influencing the NT-proBNP levels in these patients.

Further analysis revealed that in Group 2, a higher percentage of newborns presented with low birth weight as compared to those in Group 1 (Table 5), suggesting potential implications of elevated NT-proBNP levels associated with SARS-CoV-2 infection on foetal health.

Using the z-test for binomial proportions, this difference in newborn outcomes between the two groups was statistically significant (*p* < 0.05), as shown in Table 6.

Regarding the gender of the newborns analysed based on maternal COVID-19 infection and NT-proBNP values between the studied groups, our study showed no statistical significance between male or female newborns (Table 7). This provides valuable information and attributes the intrauterine growth restriction results strictly to associating SARS-CoV-2 infection and higher levels of NT-proBNP with pregnancy.

The complications associated with the COVID-19 viral infection are linked to the increased occurrence of caesarean sections in Group 2 (Table 8). Because of the virus’s possible health complications, many healthcare providers had to change their approach. When the mother’s health was at risk, an emergency caesarean section became necessary. This surgical intervention was performed as a rapid response to decrease stress on the maternal organism, with the safety of both the mother and the foetus being prioritised. The trend towards caesarean sections in this group highlighted the important decisions that medical practitioners had to make in the face of the pandemic’s exceptional problems.

As for the influence of the severity of the COVID-19 viral infection and the values of NT-proBNP associated with the newborn outcome, we divided our studied patients into four groups based on SARS-CoV-2 symptoms and severity of the disease, as presented in Table 9. Our results clearly suggest that the more severe the infection and symptoms, the more likely patients will have a higher NT-proBNP value. Also, the newborn weight and Apgar score were influenced by the severity of the disease, indicating a high risk for intrauterine growth restriction and premature birth.

## 4. Discussion

The present study, considering NT-proBNP variations in pregnant patients with and without SARS-CoV-2 infection and implicating the consequences for the newborn, provides important data that can influence the outcome, evolution, and management of the cases. Our study evaluated the influence of NT-proBNP values in pregnant patients with or without previous or active SARS-CoV-2 viral infection and its impact on the newborns’ birth weight and Apgar score.

There are various possible causes for elevated NT-proBNP levels during the peripartum period. This finding is consistent with previous research in healthy pregnant women, which found that NT-proBNP levels stay normal throughout pregnancy with a brief spike during labour and delivery [17,18]. In order to rule out heart failure, NT-proBNP readings must be interpreted as a continuous variable with “normal” values such as 70 pg/mL [19].

In pregnancy, an increased NT-proBNP level may indicate subclinical, impaired heart function. NT-proBNP levels were 81 ng/mL before birth and 165 ng/mL after delivery in a small study of 88 pregnant women (mean age: 30.5 years and gestational age: 39.5 weeks (95% CI: 35–42)). Other studies have found levels of up to 700 ng/mL [20]. NT-proBNP has been linked to pregnancy complications [21]. In the pathogenesis of gestational hypertension, increased blood pressure is associated with an increased cardiac burden, excessive ventricular volume and pressure overload, and increased wall tension or stretch stimulation of ventricular myocytes, resulting in increased NT-proBNP secretions. NT-proBNP also lacked diagnostic efficacy for gestational hypertension, presumably because it originates from both the heart and the placenta [22]. There is no link between NT-proBNP levels and parity, labour time, or offspring birth weight; however, a study by Lev-Sagie et al. found an increase in NT-proBNP levels in women who received an epidural, pethidine, or inhaled nitrous oxide [23]. Some studies link the NT-proBNP pathway to total peripheral resistance. According to this, peripheral resistance in physiological pregnancies is stronger in the first trimester and diminishes as pregnancy progresses towards 20–24 gestational weeks, with a modest rise in the final weeks of gestation [24].

In patients with SARS-CoV-2, NT-proBNP levels are frequently elevated, and this is strongly linked to myocardial damage and mortality [25].

Multiple pathophysiological processes may be responsible for the elevated NT-proBNP levels during the COVID-19 infection. Inflammation may play a role in higher levels of circulating natriuretic peptides [26]. The presence of large levels of natriuretic peptides has been linked to a decrease in cardiac function in SARS-CoV-2 [27]. Pregnancies with intrauterine growth restriction have NT-proBNP levels comparable to hypertensive and unaffected pregnancies. An NT-proBNP with a value higher than 136 pg/mL has a significant positive predictive value for delivery within 10 days [28].

In pregnancies with chronic hypertension, NT-proBNP levels can be used to predict low birth weight [29]. Foetuses with an IUGR history have a higher mortality rate, greater perinatal issues, and sequelae, such as impaired visual, auditory, and executive functioning; developmental delays; psychological and behavioural problems; cardiovascular and pulmonary conditions; and resulting socioeconomic burdens [30,31]. Many other aspects, including the influence of sex-specific placental features and their reactions to the harsh prenatal environment, have yet to be properly investigated. IUGR pregnancy has a complex aetiology [32]. Preterm births and intrauterine development restrictions occurred in pregnancies complicated by COVID-19. Pregnant women with COVID-19 should be closely examined clinically, using ultrasonography to diagnose these diseases [33]. However, it is uncertain if COVID-19 is the direct cause of preterm labour and delivery; viral infection during pregnancy can create an abnormal response to an opportunistic bacterial infection, which can contribute to preterm labour and delivery [34]. Despite the virus’s low vertical transmission, those who had COVID-19 during pregnancy are more likely to have a placental infarction because of heightened pro-inflammatory cytokines and von Willebrand factors during pregnancy and SARS-CoV-2 viral infection. Infarcts in the placental circulation can cause placental insufficiency, decreased oxygen and nourishment supply, foetal growth restriction, delayed foetal brain development, an increased chance of premature birth, and even newborn mortality [35,36,37,38].

According to one study, the pooled mean birthweight was 3144.71 g. COVID-19 is an acute infection; thus, if it develops near delivery, it is unlikely to affect birth weight. However, for early pregnancy infections and women with chronic hypoxia, a growth ultrasound is indicated to assess the risk of intrauterine development restriction [39]. Premature and severely LBW babies, particularly those born before 28 weeks, have decreased by 70 to 90 percent in several parts of the world, according to studies from Denmark and Ireland [40,41].

According to the findings of another study, the pooled fraction of positive SARS-CoV-2 in placental specimens was nearly twice that of other specimens. ACE2 is highly expressed at the maternal–foetal interface, which includes the placenta and decidua [42]. As a result, it is possible that the virus will penetrate and invade the placenta, increasing placental permeability. It may result in placental insufficiency and several of the obstetric problems documented in COVID-19-infected women, such as abortion, LBW, or preterm birth [43].

The broad prospect of medical research and clinical studies repeatedly implies that pregnant women have a higher sensitivity to various infections. This sensitivity can be related to a number of physiological and immunological changes that occur during pregnancy. Given this established understanding, especially in light of the recent global health crisis caused by the SARS-CoV-2 virus infection, it is crucial to emphasise the potential risk that pregnant patients may face. Furthermore, not only pregnant patients deserve this attention but also their newborns, who should be recognised as a population that could be particularly vulnerable to the COVID-19 viral infection due to common physiological links and early exposure. As a result, it is critical for healthcare providers to consider these patients while developing protective methods and guidelines [44].

While our primary goal was to determine whether or not the viral infection with COVID-19 increased the stress of the cardiovascular system and increased NT-proBNP values, which automatically increase the risk of heart failure in general, as well as its effect on the newborn’s weight and Apgar score, it is also important to note that it had a significant influence on the method of delivery, which was mostly caesarean section.

Our findings highlight the importance of monitoring a pregnant patient’s cardiovascular status by measuring the level of NT-proBNP, especially if the pregnancy is associated with SARS-CoV-2 viral infection, because of the extended risk to the cardiovascular system and its implications, as well as the influence on the newborn.

### Strengths and Limitations

This study had various strengths that contributed to its scientific validity and rigour, including the careful selection of cases, a well-executed and planned study design, and a focus on a vulnerable group of patients, pregnant women with or without a history of SARS-CoV-2 viral infection during pregnancy. Furthermore, our findings provide important information and insights for prenatal treatment and risk factor identification in pregnant patients and their newborns. Finally, NT-proBNP in pregnant women, particularly those with COVID-19 viral infection, has received insufficient attention, and our findings can contribute to this critical topic. All the strengths mentioned above add to the significance and validity of the study’s findings.

While this study has considerable strengths, certain inherent constraints that may impair its generalisability must be acknowledged. First and foremost, the study’s temporal constraints, characterised by its relatively short duration, may potentially limit its complete depth. Furthermore, its single-centre design prohibits complete coverage of the varied demographics of pregnant patients, thereby narrowing the scope of the study. Another relevant limitation is the sample size; the small number of patients investigated may not provide the validity that a larger, more diverse cohort would. These findings emphasise the significance of exercising caution when interpreting the findings and give a direction for future research into the interaction between NT-proBNP levels in pregnant patients with or without COVID-19 viral infection and its implications for the newborn.

Our goal is to continue this study within our esteemed institution while also seeking collaboration with other esteemed institutions and noteworthy scholars in this field. We are sure that broadening the breadth of our research is critical to strengthening the legitimacy of our preliminary findings. To provide a more comprehensive understanding of the consequences of NT-proBNP values and their significance in assessing newborn risks among pregnant patients with or without SARS-CoV-2 viral infection, we must expand our study population. Furthermore, conducting parallel analyses across several research institutions will not only increase the validity of our findings but will also assure their applicability in a variety of clinical contexts.

## 5. Conclusions

Elevated NT-proBNP had an unfavourable implication on the newborn birth weight and Apgar score. Elevated levels of NT-proBNP were found in pregnant patients who suffered from COVID-19 during pregnancy, as opposed to those without the infection, especially those who manifested a more severe form of disease. In conclusion, we consider that NT-proBNP testing in pregnant patients, especially those with COVID-19 infection, can be relevant and be used as a predictive marker to be taken into consideration when it comes to management, outcome, and treatment regarding pregnant patients and their newborns.

## Figures and Tables

**Table 1:** Age characteristics of pregnant patients stratified by SARS-CoV-2 infection status.

Group	Number of Patients	Mean Age	SD	*p* Value
1 (no SARS-CoV-2)	99	29.2 ± 6.1 years	6.1	0.7421
2 (with SARS-CoV-2)	87	29.5 ± 6.3 years	6.3	

**Table 2:** Demographic and clinical characteristics of individuals based on SARS-CoV-2 status.

Feature/Group	No SARS-CoV-2 (*n* = 99)	With SARS-CoV-2 (*n* = 87)	*p* Value
**Mean age**	29.3 ± 6.2 years	29.4 ± 6.1 years	0.912
**BMI (kg/m^2^)**	24.8 ± 3.7	24.7 ± 3.8	0.856
**Number of previous pregnancies**	1.5 ± 0.8	1.6 ± 0.7	0.368
**Gravidity**	2.0 ± 0.9	2.0 ± 1.02	>0.999
**Parity**	1.2 ± 0.7	1.3 ± 0.6	0.300
**Number of ANC visits**	6.7 ± 2.0	6.6 ± 2.1	0.740
**Education level (%)**			
Primary	10%	11%	0.645
Secondary	45%	44%	0.762
Tertiary	45%	45%	1
**Living environment (%)**			
Urban	60%	59%	0.757
Rural	40%	41%	0.762
**Occupation (%)**			
Unemployed	26%	33%	0.035
Skilled worker	31%	41%	0.002
Professional	43%	26%	<0.001

**Table 3:** Associated comorbidities in pregnant patients with and without SARS-CoV-2 infection.

Associated Pathologies	Group 1 (*n* = 99)	Group 2 (*n* = 87)	*p* Value
Hypothyroidism	5 (5.05%)	3 (3.44%)	0.435
Obstructive sleep apnoea	1 (1.01%)	3 (3.44%)	0.255
Thrombophilia	11 (11.11%)	15 (17.24%)	0.23
Bronchial asthma	3 (3.03%)	0 (0%)	<0.001
Chronic viral infections	11 (11.11%)	4 (4.59%)	0.103
Autoimmune diseases	7 (7.07%)	2 (2.29%)	0.131

**Table 4:** Comparison of average NT-proBNP levels in pregnant patients with and without SARS-CoV-2 infection.

	Group 1 (No SARS-CoV-2)	Group 2 (With SARS-CoV-2)
Average NT-proBNP level (pg/mL)	88.7 ± 24.8	542.3 ± 429.5
*p* value *t*-test	<0.05	

**Table 5:** Incidence of low birth weight in newborns based on maternal SARS-CoV-2 infection status.

Group	Percentage of Newborns with Low Birth Weight	*p* Value
1 (no SARS-CoV-2)	12%	<0.0001
2 (with SARS-CoV-2)	51%	

**Table 6:** Neonatal characteristics based on maternal SARS-CoV-2 infection and NT-proBNP levels.

Neonatal Characteristics	Group 1 (No SARS-CoV-2 and Normal NT-proBNP)	Group 2 (With SARS-CoV-2 and Elevated NT-proBNP)	*p* Value
Average birth weight (grammes)	3200 ± 400	2800 ± 180	<0.0001
Average gestational age	39.2 ± 1.3 weeks	37.8 ± 1.2 weeks	<0.0001
Average Apgar score at 5 min	9 ± 1	7 ± 1.5	<0.0001

**Table 7:** Neonatal characteristics based on maternal SARS-CoV-2 infection, NT-proBNP levels, and gender of the newborns.

Neonatal Characteristics	Group 1 (No SARS-CoV-2 and Normal NT-proBNP)		*p* Value	Group 2 (With SARS-CoV-2 and Elevated NT-proBNP)		*p* Value
Gender	Male (*n* = 65) 65.65%	Female (*n* = 34) 34.34%		Male (*n* = 39) 44.82%	Female (*n* = 48) 55.17%	
Average birth weight (grammes)	3300 ± 300	3200 ±4 00	0.1646	2780 ± 200	2700 ± 280	0.1041
Average gestational age	38.2 ± 2.3 weeks	38.2 ± 2 weeks	>0.9999	37.3 ± 0.7	37.5 ± 1.5	0.4724
Average Apgar score at 5 min	9 ± 1	9 ± 1	>0.9999	7 ± 1.5	7 ± 1	>0.9999

**Table 8:** Distribution of patients from groups 1 and 2 according to the way of birth.

	No SARS-CoV-2 (*n* = 99)	With SARS-CoV-2 (*n* = 87)	*p* Value
Caesarean section	40 (40.4%)	61 (70.11%)	<0.001
Natural birth	59 (59.59)	26 (29.88%)	<0.001

**Table 9:** Distribution of NT-proBNP values, SARS-CoV-2 form of disease, and newborn outcome.

	Asymptomatic	Mild	Moderate	Severe
NT-proBNP (pg/mL)	242.3 ± 329.5	532.3 ± 419.5	538.3 ± 412.5	742.3 ± 124.5
Neonatal characteristics				
Average birth weight (grammes)	2300 ± 430	2500 ± 400	200 ± 120	1820 ± 340
Average gestational age	38 ± 2	36 ± 5	35 ± 5	28 ± 8
Average Apgar score at 5 min	8 ± 1	7 ± 4	7 ± 3	5 ± 3

## Data Availability

The datasets used and/or analysed during the current study are available from the first author.
